# Avocado Oil: Characteristics, Properties, and Applications

**DOI:** 10.3390/molecules24112172

**Published:** 2019-06-10

**Authors:** Marcos Flores, Carolina Saravia, Claudia E. Vergara, Felipe Avila, Hugo Valdés, Jaime Ortiz-Viedma

**Affiliations:** 1 Departamento de Ciencias Básicas, Facultad de Ciencias, Universidad Santo Tomás, Avenida Carlos Schorr 255, Talca 3473620, Chile; claudiavergarava@santotomas.cl; 2 Escuela de Nutrición y Dietética, Facultad de Salud, Universidad Santo Tomás, Avenida Carlos Schorr 255, Talca 3473620, Chile; 3 Escuela de Nutrición y Dietética, Facultad de Ciencias de la Salud, Universidad de Talca, Talca 3460000, Chile; favilac@utalca.cl; 4 Centro de Innovación en Ingeniería Aplicada, Departamento de Computación e Industrias, Universidad Catolica del Maule, Avenida San Miguel 3605, Talca 3480112, Chile; hvaldes@ucm.cl; 5 Department of Food Science and Chemical Technology, Faculty of Chemical and Pharmaceutical Sciences, University of Chile, Santos Dumont 964, Santiago 8380494, Chile

**Keywords:** avocado oil, oil extraction, antioxidants compounds, fatty acid profile

## Abstract

Avocado oil has generated growing interest among consumers due to its nutritional and technological characteristics, which is evidenced by an increase in the number of scientific articles that have been published on it. The purpose of the present research was to discuss the extraction methods, chemical composition, and various applications of avocado oil in the food and medicine industries. Our research was carried out through a systematic search in scientific databases. Even though there are no international regulations concerning the quality of avocado oil, some authors refer to the parameters used for olive oil, as stated by the Codex Alimentarius or the International Olive Oil Council. They indicate that the quality of avocado oil will depend on the quality and maturity of the fruit and the extraction technique in relation to temperature, solvents, and conservation. While the avocado fruit has been widely studied, there is a lack of knowledge about avocado oil and the potential health effects of consuming it. On the basis of the available data, avocado oil has established itself as an oil that has a very good nutritional value at low and high temperatures, with multiple technological applications that can be exploited for the benefit of its producers.

## 1. Introduction

Avocado (*Persea americana Mill.*) is a fruit native to Central America, grown in warm temperate and subtropical climates throughout the world. The pulp of this fruit contains about 60% oil, 7% skin, and approximately 2% seed [1,2]. The main producers of avocado oil in the world are New Zealand, Mexico, the United States, South Africa, and Chile [3]. Avocado oil has sparked a growing interest in human nutrition, food industry, and cosmetics. The lipid content, mainly of monounsaturated fatty acids, is associated with cardiovascular system benefits and anti-inflammatory effects [4,5].

There are no internationally defined parameters for avocado oil. The values that are commonly used are those recommended for olive oil. The quality standard for olive oil is available in the Codex Alimentarius and the International Olive Oil Council (IOC) [6].

Woolf et al. [7] proposed a classification for avocado oil based on its extraction method and fruit quality. Avocado oil of a higher quality, “extra virgin”, corresponds to that produced from high-quality fruit, extracted only with mechanical methods, using a temperature below 50 °C and without the use of chemical solvents. “Virgin” avocado oil is produced with fruit of a lower quality (with small areas of rot and physical alterations), extracted by mechanical methods, using a temperature below 50 ° C and without the use of chemical solvents. “Pure” avocado oil is a type of oil for the production of which the quality of the fruit is not important; it is a bleached and deodorized oil, infused with the natural flavor of herbs or fruits. Finally, “mixed” avocado oil is combined with olive, macadamia, and other oils. Therefore, it presents sensory and chemical characteristics that are variable.

The Mexican norm [8] states that the “crude oil of avocado” is a slightly amber-colored fatty liquid, obtained by physical extraction of the pulp and the seed of the fruit (*Persea americana*). “Pure” edible avocado oil is a product with at least 98.5% refined avocado oil.

**Figure 1:**
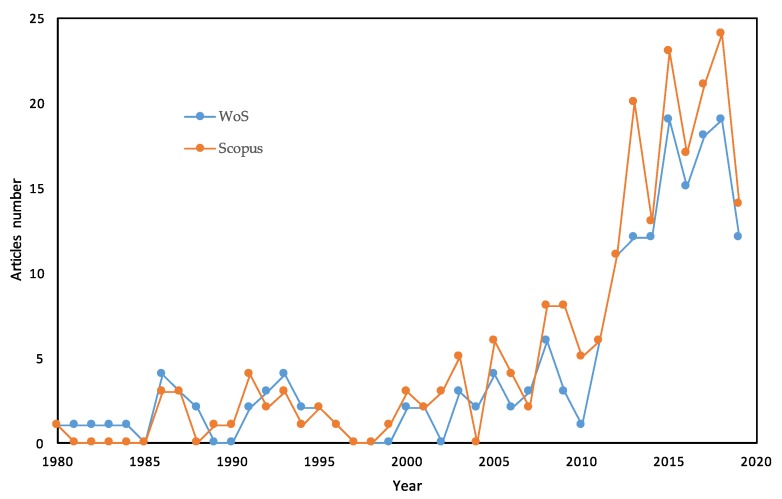
Graphic of articles (topic: “avocado oil”) from 1980 to 2019 in the Web of Science (WoS) and Scopus.

In this work, a systematic review of the literature was carried out to collect, select, evaluate, and summarize all available evidence regarding the processes and properties of avocado oil. The research question was: What are the most published topics on avocado oil? The answer to this question allowed us to include topics, such as: extraction methods (i.e., cold pressed method, ultrasound-assisted aqueous extraction method, supercritical CO_2_ method, CO_2_ subcritical method, enzymatic extraction, and solvent extraction), procedures of conservation, contamination/adulteration, technological applications, composition (characteristics according to the variety and origin of the fruit, physicochemical characterization, avocado seed oil, and comparison with other oils), and biological effects (human health effects and experimental studies in animals). Figure 1 shows the exponential increase of scientific interest in avocado oil. On the topic, “avocado oil” (from 1980 to date), 180 and 224 articles have been published in the Web of Science (WoS) and Scopus, respectively.

The purpose of this research is to produce a complete profile on avocado oil, including extraction methods, physicochemical characteristics, nutritional properties, as well as various applications in the food and medicine industries. Avocado oil has proven to be a vegetable oil with a composition of major and minor components that are highly appreciated by the population, either at low or high temperatures, with multiple technological applications.

## 2. Extraction Methods for Avocado Oil

The information presented in this section focuses on the process efficiency, improvement of production performance, and product quality as well as applications in the food industry.

Considering the high humidity percentage of avocado (around 70 to 80%), the influence of the pulp drying method prior to oil extraction has been studied [9]. The quality parameters (peroxide value, iodine value, amount of oleic acid, refractive index, electrical conductivity, content of carotenoids, chlorophyll, phenolic compounds, and antioxidant activity) have shown better results when the pulp is dried at 60 °C under vacuum, and the extraction is performed by the Soxhlet method. Meanwhile, the bioactive compounds were best preserved when the avocado pulp was dried at 60 °C with air ventilation and mechanical pressing [10]. On the other hand, avocado pulp oil, pressed and dried in a microwave, presented a better quality—determined by the acidity index, peroxide index, and oxidative stability—when compared with oil obtained by extraction with ethanol. The composition of fatty acids did not differ significantly when analyzing oil obtained by drying under microwaves or in a drying oven with forced air circulation [11]. According to Chimsook and Assawarachan [12], the studied drying method of the avocado pulp, prior to the extraction of the oil, does not significantly influence the composition of fatty acids. However, changes were determined in the antioxidant activity and vitamin E content of cold-pressed avocado oil (from Thailand). Higher antioxidant activities and a higher vitamin E content were observed in oil, the pulp of which was dried with hot air, when compared to oils obtained by an air-dried and vacuum process. This study is consistent with the fact that oils from the *fortune* avocado variety, obtained by the pulp drying lyophilization method, resulted in lower concentrations of α-tocopherol, squalene and β-sitosterol, as well as higher relative concentrations of campesterol and cycloartenol acetate, compared to oils obtained through hot air-drying processes [13].

### 2.1. Cold Pressed Method

According to the CODEX STAN 19-1981 [14], the method of extraction of edible vegetable oils is characterized by mechanical procedures, for example, extrusion or pressing, without the application of heat. In addition, the oil can only be purified by washing, sedimentation, filtration, and centrifugation.

In the cold pressing method, oil recovery is only obtained from the parenchyma cells of the pulp; its rupture begins in the first stages of grinding and it can be seen that the idioblastic cells (oil carriers) remain intact during the process of extraction. The extraction yield increases when the pulp is beaten at 45.5 °C for 2 h [15]. In this method, a lower extraction yield is obtained, although with higher concentrations of α-tocopherol and squalene, as well as lower contents of campesterol and cycloartenol acetate, compared to the Soxhlet method [13]. Drying by lyophilization and subsequent extraction by the Soxhlet method allows for a better extraction performance. However, when drying by lyophilization and extracting by cold pressing, oils with a greater concentration of antioxidants, and other bioactive compounds were obtained [13].

### 2.2. Ultrasound-Assisted Aqueous Extraction Method (UAAE)

This method uses the cavitation forces produced by acoustic waves to break down the cell walls of the oil-containing cells. This process allows for the generation of an emulsion, which facilitates oil extraction. This method can be carried out using an ultrasonic bath or an ultrasonic horn transducer [16]. The high frequency ultrasound conditioning (0.4, 0.6, and 2 MHz, 5 min, 90 kJ/kg) of the avocado puree can improve the oil separation and potentially reduce the beating time in industrial processes, without affecting the quality of the oil. If this treatment is applied after shaking, the extractability of the oil increases by between 2% and 5%. The oils obtained from sonicated purees showed free fatty acids (FFA) and peroxide values below the levels of industrial specification (peroxide less than 20 meqO_2_/kg) and an increase in total phenolic compounds after a 2 MHz treatment [17]. The ultrasound-assisted aqueous extraction (UAAE) of low virgin avocado oil in FFA, considered as virgin avocado oil, is that obtained by mechanical or natural means at low temperatures (<50 °C) and without chemical refining [7]. The optimal UAAE parameters to produce the highest extraction of virgin avocado oil was 6 mL/g water-dried pulp powder, 30 min of sonication time at 35 °C. The sonicated virgin avocado oil was lighter and had a higher level of unsaturated fatty acids, compared to the avocado oil extracted by the Soxhlet method [16].

### 2.3. Supercritical CO_2_ Method

This method of extraction is based on the use of supercritical fluids, substances that are, in certain circumstances, in a state in which they have intermediate properties between liquid and gas. Supercritical CO_2_ (scCO_2_) is a totally innocuous gas, which becomes a powerful solvent under conditions of pressure and at a temperature above its critical point [18].

Extraction with scCO_2_ presents a higher performance at a pressure of 400 bar. The use of ethanol as a co-solvent favors the extraction of residual oil, benefiting the extraction of a fraction enriched in tocopherols [19].

Some authors [20,21] proposed the combined extraction of avocado oil and active compounds present in peppers (capsanthin) and tomatoes (lycopene) using scCO_2_ in order to enrich the avocado oil. For this, a fixed bed extractor was used, where the lipids and the desired active ingredient were subjected to the extraction process, simultaneously with scCO_2_. First, both the scCO_2_ and the oil extracted from the avocado passed through the avocado bed and then through the second bed, where the plant component to be co-extracted was found. The lipids obtained in the first chamber served as a co-solvent with scCO_2_ for extraction in the second chamber. In the case of the simultaneous extraction of edible avocado oil and the capsanthin (carotenoid) of red pepper, the higher concentration of oil improved the extraction yield of capsanthin. However, a less concentrated extract was obtained, since the carotenoid was diluted in the product. In the case of the extraction of avocado oil rich in lycopene, the extraction yield of lycopene increased as the proportion of avocado in the first extraction chamber increased, being the best condition for the extraction of lycopene present in tomato pomace at 400 bar and 50 °C. 

Restrepo et al. [22] evaluated the quality of avocado oil extracted by Soxhlet, cold pressed, and scCO_2_ methods, determining the quality of the oil in terms of free fatty acid titration, peroxide index, iodine index, saponification, and specific gravity, according to the American Oil Chemists’ Society (AOCS) standards. Extraction with supercritical fluids was the technique by which the highest yields and quality were obtained. Oils extracted by scCO_2_ were characterized as possessing a lower acidity index (0.48%), low oxidation of unsaturated fatty acids (16.87 meqO_2_/kg) and higher iodine index (80.18 cgI2/g), when compared with the other methods. In addition, extraction by cold pressing showed better results in terms of vitamin E content.

Regarding extraction by pressurized fluids, the extraction with the liquefied gas of compressed oil (LPG), constituted by a mixture of propane, n-butane, isobutane, ethane, and other hydrocarbons, showed a higher oil extraction performance in less time and with a lower solvent consumption than the scCO_2_ method. On the other hand, the oil obtained by compressed LPG presented higher concentrations of Stigmasterol, licopersene, palmitic acid, oleic acid, and linoleic acid. However, scCO_2_ provided a higher yield in terms of antioxidant activity, which was determined by means of the 2,2-Diphenyl-1-picrylhydrazyl (DPPH) radical assay [23].

### 2.4. CO_2_ Subcritical Method

Extraction with sCO_2_ operates under the same principle as the scCO_2_ extraction, but with a temperature below 31.1 °C and CO_2_ pressure of 72.9 bar [24].

In this part of the review, a comparison of the physicochemical properties of avocado oil, extracted through sCO_2_, UAAE, and conventional solvent (AOAC 920.39 [25]) will be analyzed. Extraction with sCO_2_ was performed at 27 °C and 68 bar CO_2_, UAAE was performed with 60 mL of distilled water, an ultrasonic power of 240 W and a frequency of 40 kHz for 30 min at 35 °C, followed by a final pressing. Compared to solvent extraction, oils extracted using sCO_2_ and UAAE had higher iodine index values but lower melting points, determined by slip, free fatty acid content, and saponification index values. The oils extracted by sCO_2_ and UAAE have a clear color and higher levels of unsaturated fatty acids than the oil extracted with hexane. Regardless of the extraction method, the main fatty acids in avocado oils were oleic and palmitic acids, while the main triacylglycerols in avocado oils were palmitoyl-dioleoyl-glycerol (POO; 22.48–23.01%) and palmitoyl- Oleoyl-linoleoyl-glycerol (POL; 17.64–18.23%) [24].

### 2.5. Enzymatic Extraction

In order to improve the performance of extraction by centrifugation, the incorporation of enzymes, such as pectinases, α-amylase, proteases, and cellulase, to avocado paste have been considered. The yield varies depending on the concentration and type of enzyme used and the reaction time and percentage of water used. It is emphasized that this method improves oil by up to 25 times, in comparison with the performance of a non-enzymatic centrifugation [26].

### 2.6. Solvent Extraction

Reddy et al. [27] compared four extraction methods to produce avocado oil (*Hass* and *Fuerte* variety). They analyzed: (1) the extraction with traditional solvent using Soxhlet (5.0 g of dried avocado sample with 250 mL of hexane for 24 h); (2) Ultrasonic Soxhlet extraction (5.0 g of dry avocado sample, sonicated in a water bath at 60 °C, with hexane as the solvent, for 1 h); (3) Soxhlet extraction, combined with a microwave treatment (avocado paste 5 mm thick, extended in the rotating plate of a domestic microwave oven, heated to the maximum power for 11 min, with 5 g of the resulting mass subsequently extracted by means of the Soxhlet method with hexane); and (4) extraction with supercritical fluid (Argon and scCO_2_ used as extraction fluids, extractions performed for 2 h, with a fluid flow rate of 2.8–3.5 mL/min). The traditional Soxhlet extraction method yields the most reproducible results, whereas the microwave extraction showed a higher extraction yield and higher fatty acid content (69.94%).

Meyer and Terry [28] performed a sequential extraction and quantification of fatty acids and avocado sugars. The average oil yield using Soxhlet extraction, with ethanol as the solvent, was significantly higher than the oil obtained by homogenization with hexane, and the fatty acid profiles for the two methods were similar. As the maturity of the fruit increases, the extraction of oil is improved. After lipid removal, methanolic extraction was superior in terms of the sucrose and perseitol obtained, compared to extraction with 80% ethanol (*v*/*v*). The extraction of mannoheptulose was not affected by any of the solvents used.

The yield of avocado oil extraction has been assessed, comparing four extraction methods using solvents of different polarities. The extraction was performed using the Soxhlet method, with (1) petroleum ether, (2) homogenization with petroleum ether, (3) homogenization with a mixture of chloroform/methanol (2:1 *v*/*v*), and finally (4) extraction with chlorine-naphthalene and ball milling. It was determined that methods that only use petroleum ether as an extraction medium presented lower yields (6–9% less) than the last two methods. Saponifiable residues were lower when the method using the chloroform/methanol mixture was employed. However, this method did not completely eliminate the residual oil from the fruit [29].

Ortiz-Moreno et al. [30] analyzed the effect of four extraction methods on the chemical-physical quality of avocado oil, namely, (1) the method of microwave extraction and manual pressing, (2) extraction with hexane using the Soxhlet methodology, (3) microwave extraction, combined with the Soxhlet methodology, using hexane as the solvent, and (4) extraction with acetone. The method with the highest oil extraction performance was the third method. The amount of *trans* fatty acids produced by the first method was the lowest and the latter method is also the one that generates the least physicochemical alterations.

When analyzing the effect of the drying method and avocado oil extraction process, ripe fruit, independent of the drying method, presents a higher extraction performance than immature fruit. This is influenced by the enzymatic degradation of the cell wall of the parenchyma during maturation. Freeze drying improves the amount of oil extracted for the scCO_2_ extracts and, to a lesser extent, for the hexane extracts. Extraction with hexane has been shown to have a higher oil extraction yield than scCO_2_ due to the lower degree of selectivity of this solvent, which completely penetrates the plant material [31].

Regarding the performance of the avocado oil extraction process, methodologies have been proposed for developing countries. One is carried out with boiling petroleum ether (30–60 °C) and another extraction with distilled water (avocado paste, diluted at a ratio of 3:1 and 5:1 (*w*/*w*), heated in a water bath of 75 to 98 °C, with subsequent centrifugation. In the extraction methods, calcium chloride, sodium chloride, calcium carbonate and calcium sulfate were used as extraction aids. The presence of inorganic salts at a low concentration improves the extraction performance, provided that it does not exceed 5%; otherwise, it has an adverse effect. The most efficient extractions were obtained with a water/avocado ratio of 5:1, pH of 5.5 and centrifugal force of 12,300× *g*, with the addition of 5% calcium carbonate or calcium sulfate. At higher heating temperatures (75–98 °C), the oil release time decrease. In addition, the gravity sedimentation for four days at 37 °C, followed by centrifugation, improves the oil extraction performance [32].

Considering the use of organic solvents in avocado oil extraction processes could alter the quality of fatty acids by inducing the formation of *trans* isomers. Ariza-Ortega et al. [33] proposed the application of infrared spectroscopy by Fourier transform (FTIR) to study trans fatty acids in the avocado oils of the *Hass*, *Fuerte*, and *Criollo* varieties. For this, oil extraction was performed by centrifugation at 40 °C and extraction with hexane at 70 °C for 4 h. The method using centrifugation did not increase the deterioration of fatty acids. A strong band at 723 cm^−1^ was documented, which is attributable to the cis functional groups, where the green color was maintained. On the other hand, the infrared spectroscopy with Fourier transform (FTIR) analysis identified an absorption band, located at 968 cm^−1^, which is associated with fatty acids, with *trans* isomerism for the *Fuerte* variety extracted with hexane.

## 3. Procedures for the Conservation of Avocado Oil

The conservation of oils is a necessary issue to address, since it allows for increasing the useful life of the products. One of the efforts made to improve the conservation of avocado oil has been the use of physical techniques, such as the electric field.

The electric field (voltage 9 kV cm^−1^, frequency 720 Hz, time of 5 and 25 min) allows the polyphenol oxidase enzyme present in the avocado pulp to be inactivated, preserving the components present in the avocado oil. The modifications in the quality of the refined oil (established according to the acidity index, peroxides, and iodine) are minimal, considering the electric field method as an alternative for the addition of synthetic antioxidants [34].

The oxidative stability (determined by finding the antioxidant activity reducing ferric ion, FRAP), during the storage of cold-extracted avocado oil in the presence of the oleoresins of *Capsicum annuum L*. (vegetable material rich in carotenoids), was assessed. It was determined that the optimal extraction of carotenoids was at a concentration of 1:3 (*w*/*v*: *Capsicum annuum* L/avocado oil) for 48 h in darkness at room temperature. The behavior of the oil under stronger conditions (45 °C, 30 days) showed the following characteristics: (1) the extracts were stable to lipid oxidation, with a Totox index total value of 27.34, (2) 85.6% of carotenoids were conserved, (3) 80.66% of the antioxidant activity was retained, and (4) there was a color change (ΔE) of 1.783. The oleoresins obtained by extraction with avocado oil can be considered as an economic and sustainable alternative for the extraction of carotenoids, with a good oxidative stability, compared with organic solvents [35].

## 4. Use of Analytical Techniques in the Quantification, Adulteration, and Contamination of Avocado Oil

There is growing interest among consumers in accessing quality and authentic products. Vegetable oils can suffer from contamination and/or adulteration, which causes the product to have components not specific to the oil. It is here that the development, implementation, and application of analytical technologies are very useful.

The components present in avocado oil, such as fatty acids and phytosterols, have been quantified, mainly by gas chromatography, coupled with a flame ionization detector (GC-FID). In addition, techniques, such as ultra-high-performance liquid chromatography (UHPLC), coupled with mass spectrometry (UHPLC-MS) or a photodiode array detector (UHPLC-PDA), as well as Inductively Coupled Plasma Mass Spectrometry (ICP-MS), have been used for the identification and/or quantification of analytes, such as polyphenols, squalene and minerals, respectively [36,37]. Other analytical techniques have been used for the qualitative determination of the components present in avocado oil, including ^13^C nuclear magnetic resonance spectroscopy (NMR), which has been used for the identification of its major components, including fatty acids [38]. At the same time, ^1^H Nuclear magnetic resonance spectroscopy (^1^H-NMR) has been used for the detection of the minor components present in other vegetable oils [39]. Therefore, the development of new analytical methodologies for quantifying the analytes present in avocado oil represents a major challenge.

Rohman et al. [40] studied the purity of avocado oil, adulterated with palm oil and canola oil, through FTIR, combined with chemometric techniques. FTIR combined with multivariate calibrations can be used to detect and quantify the adulteration of avocado oil in binary mixtures with palm oil and canola oil.

The adulteration of avocado oil with soybean oil or grape seed oil can be determined using mid-infrared spectroscopy, combined with the statistical method of partial least squares discriminant analysis. This methodology allows for a simple and fast discrimination of avocado oil in binary mixtures and Tertiary oils. The frequency selected for the authentication of avocado oil was 1500–750 cm^−1^, with a precision of 100% for the analysis of the mixture of two oils and 93.3% for the mixture of three oils [41]. 

Organophosphorus pesticides in samples of commercial avocado oil were determined using atmospheric pressure microwave-assisted liquid–liquid extraction (APMAE), with solid-phase extraction or low-temperature precipitation, as the clean-up step. The analysis was carried out by gas chromatography-flame photometric detection and gas chromatography-tandem mass spectrometry. Chlorpyrifos residues were detected in one of four samples of commercially packaged avocado oil, produced in Chile [42]. While spectroscopic techniques have focused on determining the adulteration of avocado oil with the presence of other types of vegetable oil, according to the literature reviewed here, there is a research deficiency related to the modification of the composition of avocado oil, including the study of its major components, such as triacylglycerides and/or fatty acids, in addition to its minority components, such as phytosterols, alkanes, aliphatic alcohols, polyphenols, and others. This could provide information for detecting the contamination of avocado oil with other oils of a different quality.

## 5. Technological Applications of Avocado Oil

At the industrial level, there is a constant demand for the production of healthy foods that can maintain their nutritional properties over time, as well as environmentally friendly technological solutions. Avocado oil is mainly sold for direct consumption due to its interesting contribution of fatty acids, vitamins, antioxidants, among other compounds. Efforts have been made to develop products based on avocado oil. Arancibia et al. [43] propose the development of O/W nanoemulsions using the natural emulsifiers, lecithin and synthetic tween 80, systems that improve the characteristics with respect to traditional emulsions, such as (i) increased dispersibility of water in the encapsulated oils, which generates slightly turbid emulsions and an easy production, and (ii) a good physical and chemical stability, as well as a high bioavailability of its lipid components.

Another interesting technological application for avocado oil has been the production of structured lipids. Caballero et al. [44] propose the elaboration of triacylglycerides of the MLM type, using regio-specific immobilized commercial lipases *sn-1.3*, where M corresponds to saturated medium-chain fatty acids (6–12 carbon atoms) at positions *sn1* and *sn3* of glycerol. L corresponds to saturated or unsaturated long-chain fatty acids (14–24 carbon atoms) in the *sn2* position. The increased interest in this type of lipids is due to the low caloric intake (average caloric density for this family of lipids 5 kcal/g). According to the literature, there are no negative effects associated with the ingestion of MLM lipids for both animals and humans.

Finally, avocado oil has also been used in the production of biodegradable polymers. Polyhydroxyalkanoates (PHAs) are linear polyesters, produced by a large number of bacteria under stress conditions, with different thermal and mechanical properties, which depend on their molecular structure. Flores-Sanchez et al. [45] prepared PHAs through a fermentative process using the bacterium *C. necátor* H-16 with avocado oil and fructose, as a carbon source. The highest yield in obtaining polymers was obtained when the addition of avocado oil was 20% *v*/*v*, which demonstrates the feasibility of using this oil as a renewable carbon source for the PHA production process.

## 6. Composition of Avocado Oil

There is a growing interest in avocado oil, including the determination of the composition of major and minor components. Therefore, for a total understanding of the nutritional and functional properties that this oil presents, it is important to consider the different varieties and parts of the fruit. 

### 6.1. Characteristics According to the Variety and Origin of the Fruit

Avocado is a fruit grown mainly in warm temperate and subtropical climates throughout the world, so it is interesting to study how the climate and country of origin can affect the fruit quality and therefore, the oil. Thus, the oil from the fruit of the *Hass* variety, originating from crops from Mexico, Australia, the United States, and New Zealand, was characterized by a high content of 62% lipids, of which oleic (42–51%) and palmitic (20–25%) lipids were present in a greater proportion. Among the predominant triacylglycerols were OOO (21–34%) and OOP (19–24%), where O and P denote oleic and palmitic acids, respectively. On the other hand, *Hass* avocado oil from New Zealand contained a significant amount of natural pigments and unsaturated compounds, compared to oils from Mexico, Australia, and the United States [1].

Studies carried out in South America on the analysis and characterization of avocado oil showed a high content of monounsaturated fatty acids (69.4%) and a lower amount of polyunsaturated and saturated fatty acids, which were 16.6% and 14%, respectively. These studies indicate that avocado oil has a thermal stability close to 176 °C and has a lower concentration of total phenolic compounds than olive oil. Despite this, the antioxidant activity of avocado oil is similar to that of olive oil. Olive oil has a high concentration of polyphenols, such as tyrosol and hydroxytyrosol [46].

Galvão et al. [47] analyzed the fatty acid composition of the pulp, seed, and skin oil of the *Fortuna*, *Collinson*, and *Barker* varieties, indicating that there was a small variation in the composition of monounsaturated fatty acids in the skin oil among the cultivars. However, the seed oil of the Collinson variety was the best due to the lower SFA content. The SFA content for pulp oil corresponded to 22.3, 29.4 and 41.3% in the *Fortuna*, *Collinson*, and *Barker* varieties, respectively. In this sense, it was possible to affirm that the pulp oil of the *Fortuna* and *Collinson* varieties presented a better quality, in terms of fatty acid profile, than the Barker variety.

In Mexico, avocado oil from six local creole varieties (*BTancitaro*, *Irapuato*, *Orgánico*, *Puerto*, *San José*, and *STancitaro*) were analyzed and compared with oil from the *Hass* variety. It was observed that the Mexican creole genotypes had a greater thermal stability, properties resistant to oxidation, and a greater phenolic content, in comparison with the commercial oil from the *Hass* variety. In addition, these varieties showed intense fluorescent peaks at 675 and 720 nm, as well as broad absorption bands centered at 465 and 510 nm, which can be used as an identification parameter for these oils [48].

Yanty et al. [49] indicated that the avocado oil originating from three Malaysian varieties was found in a significantly lower proportion than in the Australian *Hass* variety. In addition to being in a semi-solid form, all these oils had a higher proportion of oleic acid, although they also had different proportions of palmitic and linoleic acids. Regarding the composition of TAG for local varieties, the highest was POO, followed by POL, OOO, and PPO, while in the *Hass* variety, the distribution was OOO, followed by PPO, OOL, and POL. As a result of these different compositions in TAG, differences were found in the iodine index, melting point by slip and melting and solidification characteristics.

### 6.2. Physicochemical Characterization

Table 1 shows the composition of the common fatty acids of the oils from different varieties and origin of avocados, discussed in this work. However, only some varieties have the C6:0, C7:0, C8:0, C9:0, C10:0, C11:0, C12:0, C13:0, C14: 0, C14:1, C15:0, C15:1, C16:1, C17:0, C17:1, C19:0, C20:0, C20:1, C20:3, C20:4, C22:0, C22:1, C22:2, C23:0, and C24:0 fatty acids in a low proportion, ranging from traces (<0.06%)–3.58%. Dreher and Davenport [50] refer to the fact that the oil coming from the *Hass* variety can contain up to 71% of monounsaturated fatty acids (MUFA), 13% of polyunsaturated fatty acids (PUFA), and 16% of saturated fatty acids (SFA).

In order to study the fatty acid profile of the avocado oil from the fruits harvested and artificially ripened, the avocado oil was extracted from preserved fruit at 5 °C in a controlled atmosphere and was analyzed (4% of O_2_ and 6% of CO_2_). Postharvest ripening was stimulated in the presence of exogenous ethylene (0 or 100 ppm) at a temperature of 18 °C for 24 h and then preserved at 15 and 20 °C. It was concluded that post-harvest conservation and ripening by means of a controlled atmosphere did not have a detrimental effect on the fatty acid profile or the amount of oil obtained, when compared with the one commonly applied in the ready-to-eat market [53]. This information is very important for the fresh product industry, where avocados move long distances at low temperatures.

When carrying out the physicochemical evaluation of two Hass avocado oils sold in Chile and labeled as extra virgin, it was demonstrated that both oils presented significant differences in the content of tocopherols, total phenols, oil stability, measured as the induction time, UV absorption coefficients, peroxide index, free acidity, total chlorophyll, total carotenoids, and polar compounds. In addition, the presence of 3,5-stigmastadiene in one of the samples, a compound that has been associated with a high degree of refining or exposure to high temperatures of oils, indicated a disparity in the quality parameters and a lack of regulation of avocado oil in the local market [37].

On the other hand, monovarietal oils from the *Bacon*, *Fuerte*, *Hass*, and *Pinkerton* varieties, obtained in Spain, were compared with commercial oils originating in Brazil, Chile, Ecuador and New Zealand. The content of triacylglycerols, fatty acids, aliphatic, and terpene alcohols, desmethylmethyl, methyl and dimethyl sterols, squalene and tocopherols were determined. The main triacylglycerols were those with ECN48 (48 equivalents of carbon atoms). The oleic, palmitic, and linoleic fatty acids were the most abundant fatty acids, and the desmethyl sterols were the main quantified minor compounds. Small amounts of aliphatic and terpene alcohols were observed. The concentrations of squalene were higher in the oils of the *Bacon, Fuerte*, and *Pinkerton* varieties than in the other varieties. The most abundant tocopherol was α-tocopherol [52].

While the fruit reaches a minimum of 8% fat at the time of its extraction from the tree, during vegetative ripening, values of 20% or more are reached, depending on the variety. In this sense, it is recommended that the avocado oil industry ripen the fruit on the tree, since climacteric ripening, in comparison with commercial ripening, influences not only the increase in oil content, but also the profile of the fatty acids, increasing the amount of unsaturated fatty acids, such as oleic acid, and decreasing the amount of saturated fatty acids, such as palmitic and palmitoleic acids [56]. This situation seems controversial with respect to the previously discussed studies. However, it is well known that the composition of fruits depends on environmental and growth conditions. In addition, the quantification of the different analytes depends on the conditions of extraction, processing, and detection limits of the analytical equipment.

Martinez-Nieto and Moreno-Romero [57] have analyzed the sterol composition of the unsaponifiable matter of avocado oil in 4 varieties (*Reed*, *Bacon*, *Fuerte*, and *Hass*) by means of gas chromatography. The results showed that the *Fuerte* and *Reed* varieties contained 2% more of *β*-sitosterol than the *Bacon* and *Hass* varieties. In relation to the cholesterol content, the smallest amount was present in the *Fuerte* variety and the largest in the Hass variety. In this sense, the unsaponifiable sterol composition can be a guiding tool to determine the authenticity of oil.

Martínez-Nieto et al. [51] analyzed the composition of different fractions in an industrial extraction process using a continuous method, under conditions similar to those used in the extraction of olive oil, for ripe avocados of the *Fuerte*, *Reed*, *Hass*, and *Antillana* varieties. They concluded that it is possible to obtain a good net oil yield with the industrial equipment used prior to specific modifications in the grinding stage and in the decanter, as well as good quality parameters, such as the acidity index, peroxide index, and absorbance coefficients, for both virgin oil and refined avocado oil.

When evaluating the physicochemical characteristics of the avocado oil obtained from the *Bantul*, *Purwokerto*, and *Garut* varieties, originating in Indonesia, by extraction with solvents, the *Garut* variety presented a better quality in terms of the iodine index, which is associated with a greater amount of unsaturated fatty acids. The conjugated dienes and trienes were significantly different between the samples. The p-anisidine index did not have significant differences between the samples. The saponification index was higher in the *Purwokerto* variety, and the peroxide index was higher in the *Bantul* variety. In addition, the analysis using differential scanning calorimetry (DSC) showed that the three samples had different melting and crystallization profiles [58].

### 6.3. Avocado Seed Oil

In relation to avocado seed oil, Barrera-López and Arrubia-Vélez [59] pointed out that the Lorena variety contained about 8.47% oil, and the unsaponifiable matter was 76.9%. The phytosterols quantified in a greater proportion were ergosterol, 5α-cholestane and stigmasterol.

Avocado seed presents, in its composition, a large number of extractable polyphenols, which have attracted attention due to their high antioxidant capacity. It was determined that, with a higher power of the ultrasound (0–104 W) and increase of the temperature (20–60 °C), the polyphenol content and antioxidant capacity was increased [60].

When performing a physicochemical analysis of the seed oil of the Hass variety, cultivated in Peru and obtained by the Soxhlet method, it was found that it had a high fatty acid profile in linoleic acid (48.77%) and linolenic acid (12.17%). While the antioxidant activity, determined by the DPPH method, was low, it was higher in the saponifiable fraction than in the unsaponifiable fraction, which was attributable to the presence of polyphenols and steroids. In addition, it was determined that the quality parameters, such as acidity, peroxide, saponification, iodine, and specific gravity indexes, were similar to those for extra virgin olive oil [61].

When comparing the composition of oil from the pulp and seed of the *Fuerte* variety, cultivated in the region of Northeastern Brazil, a great difference in the lipid content between the pulp and the seed can be seen (15.39% v.s. 1.87%, dry base). It was determined that the parameters of oil quality, refractive index, gravity, and peroxide index were similar for both oils, but the iodine, acid index, and saponification index were higher in seed oil than in pulp oil. Gas chromatography showed that seed oil had a greater variety of fatty acids than pulp oil. Additionally, the fatty acid profile of the pulp was much more concentrated in monounsaturated fatty acids than that of seed, and conversely, the seed oil is much more concentrated in polyunsaturated fatty acids than pulp oil [55].

### 6.4. Comparison with Other Oils

Dubois et al. (2007) [62] compared 80 varieties of vegetable oils, including avocado oil, indicating that it was composed of more than 60% of monounsaturated fatty acids, a characteristic shared with olive oil, hazelnut, and macadamia nut profiles. In comparison with olive oil, avocado oil possessed a higher proportion of saturated fatty acids (16.4%), with a predominance of palmitic acid (15.7%), a lower proportion of monounsaturated fatty acids (67.8%), with a predominance of oleic acid 60.3% and a higher proportion of polyunsaturated fatty acids (15.2%), the most important of which was linoleic acid at 13.7%.

Additionally, similar fatty acid profiles have been published by Berasategi et al. [3], who also showed that avocado oil had a higher PUFA/SFA and higher omega-6/omega-3 ratios than olive oil.

Berasategi et al. [3] showed that the phytosterol content was higher in avocado oil (3.3 g to 4.5 mg/g of oil) than in olive oil, of which the most abundant was ẞ-sitosterol, followed by sitostanol, cycloartenol, cycloeucalenol and D7-avenasterol. The number of sterols in the avocado oil was higher, 4-demethyl-sterols being the most abundant, reaching 80% of the total fraction of sterols. This greater proportion was even maintained under drastic conditions of deterioration (180 °C). Moreover, avocado oil has a lower proportion of vitamin E, compared to olive oil. This study indicates that the thermal stability of avocado oil is similar to that of olive oil. From Table 2, it is possible to appreciate a summary of the different antioxidant components present in the avocado oils of the different varieties shown in this work.

## 7. Biological Effects

The presence of compounds with nutritional interest, such as unsaturated fatty acids (MUFA and PUFA), as well as compounds with biological activity, such as tocopherols, tocotrienols, phytosterols, carotenoids, and polyphenols, have made avocado oil of growing interest for research on the possible biological effects of avocado oil, with the aim of preventing and treating diseases through the diet of the population.

### 7.1. Human Health Effects

A study in 13 healthy adults with a habitual hypercaloric and hyperlipidic diet, where butter was replaced by avocado oil extracted at 35 °C from the pulp alone, was conducted. The incorporation of avocado oil for a period of six days reflected an improvement in the postprandial profile of insulin, glycemia, total cholesterol, low-density lipoproteins, triglycerides, and inflammatory parameters, such as C-reactive protein (CRP) and interleukin-6 [63]. Avocado pulp oil (Mexican creole genotypes) has shown anti-inflammatory activity by inhibiting the enzymes COX 1 and COX 2 in a similar way to the drug, ibuprofen, and extra virgin olive oil [48]. Additionally, when avocado oil was added to vitamin B12 skin cream preparation, it was well tolerated and had the potential for long-term topical therapy of psoriasis [64].

### 7.2. Experimental Studies in Animals

When administering avocado oil to Wistar diabetic rats for 90 days (1 gr/250 g weight), it was observed that it promoted an improvement in the functionality of the electron transport chain and decreased the generation of free radicals in the liver, attenuating the harmful effects of oxidative stress [65].

In the brain, an improvement in mitochondrial function has been observed, as well as a decrease in free radical levels, lipid peroxidation, and an improvement in the reduced/oxidized glutathione ratio. These results demonstrate that supplementation with avocado oil prevents mitochondrial dysfunction in the brain and liver of diabetic rats [66].

Ortiz-Avila et al. [67] demonstrated that supplementation with avocado oil in Wistar rats led to a reduction of the alterations in the electron transport chain at the renal level, attenuating the oxidative damage, although a protective effect on lipid peroxidation was not evidenced. 

Some authors [68,69,70] studied the relationship between avocado oil and collagen metabolism in both the skin and liver, finding that oil obtained from intact fruit (pulp and seed), refined with hexane, was associated with fibrosis in the liver, an increase in liver enzymes and consequently hepatotoxicity.

In addition to an increase in the solubility of collagen, behavior that is attributed to a decrease in the activity of lysyl oxidase was observed [66]. A similar conclusion was proposed by Lamaud et al. [71], stating that the mixture of soybean and avocado oils decreased the degree of collagen cross-linking, a process that has been associated with a delay in wound healing. However, Oliveira et al. [53] indicated that the healing activity of a semi-solid formulation of avocado oil (50/50 Vaseline) and avocado pulp oil promoted the increase of collagen synthesis and decreased the number of inflammatory cells during the process of wound healing.

Regarding the effects on cardiovascular health, it is possible to point out that the atherogenic power of avocado oil could be similar to that of olive oil and lower than that of corn and coconut oil [72]. On the other hand, an avocado oil-rich diet modifies the fatty acid content in cardiac and renal membranes in a tissue-specific manner in Wistar rats. The rise in renal arachidonic acid suggests that diet content can be a key factor in vascular responses [73].

Márquez-Ramírez et al. [74] studied the antihypertensive effect of avocado oil in rats with induced hypertension. They were subsequently treated with the administration of 1 mL of oil per 250 g of rat weight or 40 mg of losartan potassium per kg of rat weight. Avocado oil significantly decreased both systolic and diastolic pressure in hypertensive rats, but not in controls. Avocado oil mimicked the effects of the drug, losartan, on blood pressure, vascular performance, and oxidative stress.

In rats fed with sucrose, it was observed that the addition of avocado oil in the diet reduced the levels of triglycerides, VLDL and LDL, without affecting the levels of HDL. It also reduced the level of ultrasensitive CPR, indicating that the inflammatory processes associated with metabolic syndrome were partially re-established [4]. Additionally, a diet high in sucrose, which causes liver alteration in Sprague-Dawley Weaned rats, was partially reversible by the administration of avocado oil, obtained by centrifugation and extracted by solvents. This effect was similar to that of olive oil, but would not be extended to the pancreatic level [5]. In relation to insulin resistance induced by a diet high in sucrose in Wistar rats, it was possible to reduce it through the dietary addition of 5–20% avocado oil [75]. In female Wistar rats exposed to prolonged androgenic stimulation, avocado oil improved triglyceride, VLDL and HDL levels, generating a direct regulatory effect on the lipid profile [76]. The influence of avocado oil on cell damage, induced by methyl methanesulfonate (MMS) and doxorubicin, can be carried out using in vitro (V79 cells) and in vivo models (Swiss mice). Avocado pulp oil had no genotoxic effects. The oil was effective in reducing the chromosomal damage induced by MMS and doxorubicin. However, an increase in liver tissue damage was observed when evaluating a high dose of avocado oil (measured as an increase in the hepatic enzyme aspartate aminotransferase), a phenomenon attributable to the high concentration of palmitic fatty acid [77]. Table 3 shows a summary of the biological effects presented in this study.

## 8. Conclusions

While the composition and quality of the avocado oil depends on the origin, weather conditions, variety, and extraction methods, it is characterized as a mainly monounsaturated oil, with an adequate proportion of polyunsaturated fatty acids, similar to olive oil. In addition, it contains other bioactive compounds, present in the unsaponifiable fraction, such as tocopherols, polyphenols, and a remarkable proportion of phytosterols. This oil has also been shown to perform well at high temperatures. All these characteristics indicate that avocado oil has nutritional properties that are greatly appreciated by the population, even for technological applications. This makes it a product of increasing exploitation interest for producers.

This growing interest also requires a greater development of studies on the adulteration and contamination of avocado oil, as well as the further assessment of the promising biological effects of the different components present in the oil, either in animals or humans. Furthermore, considering the presented information, it is necessary to inquire into the international regulations concerning the different qualities of this oil.

## Figures and Tables

**Table 1:** Composition (%) of the common fatty acids of avocado oil.

Varieties and/or Country of Origin	Palmitic 16:0	Estearic 18:0	Palmitoleic 16:1 Ω7	Oleic 18:1 Ω9	Linoleic 18:2 Ω6	α linolenic 18:3 Ω3	Ref.
*HASS*	18,62	0,49	8,47	60,17	10,97	0,98	[51]
	13.4	0.6	3.9	65.3	15.2	1.3	[46]
	17.37 ± 0.0015	0.63 ± 0.0002	7.52 ± 0.0002	62.89 ± 0.0019	10.64 ± 0.0004	0.72 ± 0.0001	[37]
	18.17 ± 0.02	0.37 ± 0.00	4.03 ± 0.01	51.76 ± 0.04	11.12 ± 0.01	0.59 ± 0.00	[52]
	13.7 ± 1.5	-	3.4 ± 0.4	67.4 ± 3.0	14.4 ± 1.8	1.1 ± 0.01	[53] ^3^
Australia	25.63 ± 0.11	0.45 ± 0.16	7.29 ± 0.05	42.59 ± 0.16	20.87 ± 0.10	3.19 ± 0.06	[1]
México	22.59 ± 0.23	0.24 ± 0.02	11.63 ± 0.13	49.19 ± 0.57	14.72 ± 0.06	1.63 ± 0.16	
New Zealand	20.61 ± 0.16	0.30 ± 0.01	10.31 ± 0.03	50.97 ± 0.30	16.10 ± 0.11	1.72 ± 0.02	
United States	22.24 ± 0.05	0.93 ± 0.08	13.14 ± 0.01	47.69 ± 0.03	14.47 ± 0.01	1.54 ± 0.00	
*FORTUNE*	10. 75	0.48	3.14	74.32	10.03	0.85	[51]
	20.5	0.5	6.8	60.6	13.2	-	[47]
*BACON*	12.16 ± 0.04	0.38 ± 0.01	6.57 ± 0.01	61.72 ± 0.30	8.30 ± 0.02	0.44 ± 0.00	[52]
*PINKERTON*	16.93 ± 0.03	0.43 ± 0.01	7.33 ± 0.05	57.39 ± 0.18	8.25 ± 0.02	0.56 ± 0.00	[52]
*MARGARIDA*	23.66		3.58	47.20	13.46	1.60	[54]
*FUERTE*	21.312 ± 0.550	0.762 ± 0.021	2.391 ± 0.188	64.436 ± 0.666	9.147 ± 0.030	0.467 ± 0.016	[55]
	12.37 ± 0.01	0.51 ± 0.01	7.58 ± 0.00	64.62 ± 0.20	8.46 ± 0.02	0.47 ± 0.00	[52]
*BREDA*	19.9–21.3	-	2.7–7.0	57.1–64.5	10.6–11.0	0.4–0.6	[10] ^1^
CRIOLLA MEXICANA	28.12–34.48	0.23–1.07	6.64–8.5	40.73–42.72	15.52–18.88	1.51–2.14	[24] ^2^
DE MALASIA	30.37 ± 0.06	1.30 ± 0.01	5.22±0.02	43.65 ± 0.04	17.45 ± 0.04	2.03 ± 0.01	[49]
*REED*	18.18	0.40	6.56	60.25	13.03	1.40	[51]
*ANTILLANA*	18.87	0.59	4.16	63.07	11.83	1.32	[51]
VARIETY NO INDICATED	18.74 ± 0.06	0.51 ± 0.00	7.88 ± 0.01	54.40 ± 0.10	10.87 ± 0.01	0.61 ± 0.00	[3]
	12.87	1.45	3.86	57.44	18.70	0.92	[53]

^1^ composition studied under different extraction methods (cold pressed and solvents); ^2^ composition studied under different extraction methods (solvents, SCO_2_, and UAAE); ^3^ in conditions similar to natural ripening.

**Table 2:** Antioxidant compounds present in avocado oil. Concentration [=] mg × kg^−1.^

Varieties	β-Sitosterol	α-Tocopherol	γ-Tocopherol	∆5-avenasterol	Campesterol	Estigmasterol	Sitoestanol	Campestanol	Ref.
*HASS*	91.917 ± 0.027	-	-	-	6.091 ± 0.026	0.001 ± 0.001	-	-	[57]
	95.2	-	-	-	4.7	0.13 ± 0.00	-	-	[51]
	82.95 ± 0.06	86.75 ± 0.62	9.02 ± 0.09	6.63 ± 0.07	5.88 ± 0.01	-	0.46 ± 0.01	0.04 ± 0.00	[52]
*FUERTE*	94.767 ± 0.012	-	-	-	5.043 ± 0.012	0.001 ± 0.001	0.57 ± 0.04	-	[57]
	92.9	-	-	-	6.4	-	-	-	[51]
	80.56 ± 0.08	103.11 ± 6.87	20.35 ± 1.22	8.81 ± 0.03	4.62 ± 0.02	0.15 ± 0.00	-	0.04 ± 0.00	[52]
*BACON*	92.189 ± 0.012	-	-	-	6.096 ± 0.010	0.011 ± 0.001	-	-	[57]
	82.6 ± 0.03	51.90 ± 0.04	71.61 ± 0.57	9.16 ± 0.03	3.71 ± 0.01	0.40 ± 0.01	0.58 ± 0.04	0.05 ± 0.00	[52]
*REED*	94.605 ± 0.027	-	-	-	5.123 ± 0.021	0.001 ± 0.001	-	-	[57]
*ANTILLANA*	91.2	-	-	-	8.6	-	-	-	[51]
	89.3	-	-	-	10.6	-	-	-	[51]
*PINKERTON*	84.08 ± 0.08	45.62 ± 0.19	13.71 ± 0.56	5.86 ± 0.01	6.00 ± 0.01	0.11 ± 0.00	0.41 ± 0.03	0.04 ± 0.00	[52]
VARIETY NO INDICATED	-	-	-	9.42 ± 1.69	18.36 ± 1.44	1.11 ± 0.12	2.19 ± 0.22	0.43 ± 0.03	[3]

**Table 3:** Experimental studies on the biological effects of avocado.

Animal Model	Protocol Used	Conclusions	Ref.
Male Wistar rats	Daily administration of 1.0 mL/250 g of avocado oil by gavage or losartan at 40 mg/kg for 45 days.	(a) Avocado oil mimics the effects of losartan. (b) Effects of avocado oil could be mediated by decreased actions of Angiotensin-II in mitochondria. (c) The intake of avocado oil could mitigate the harmful effects of hypertension in kidneys.	[74]
Diabetic male rats Goto-Kakizaki; the control rats were Wistar males.	Daily administration of avocado oil was 1 mL/250 g of weight for a period of 3, 6 and 12 months to Winstar and Goto-Kakizaki rats.	In the brain: (a) Improvement of mitochondrial function. (b) Decreased levels of free radicals and lipid peroxidation. (c) Improvement of the reduced/oxidized glutathione ratio. (d) Prevention of mitochondrial dysfunction.	[66]
In vitro (Chinese hamster lung fibroblasts/V79 cells) and in vivo models (Swiss mice).	Swiss mice were given avocado oil in different concentrations of 250, 500, 1000 and 2000 mg/kg in an in vivo model. In vitro model was applied to different concentrations of avocado oil: 100, 200 and 400 μg/mL.	(a) Avocado pulp oil has no genotoxic effects. (b) The oil was effective in reducing the chromosomal damage induced by methyl methanesulfonate and doxorubicin. However, an increase in hepatic enzyme aspartate aminotransferase was found to be a marker of liver damage.	[77]
Wistar rats	Administration of a diet with different concentrations of avocado oil to insulin resistant rats.	(a) The dietary addition of 5–20% avocado oil can reduce glucose tolerance and insulin resistance, induced by the high sucrose diet, in Wistar rats. (b) The addition of 5–30% avocado oil reduced the body weight gain induced by the high sucrose diet in Wistar rats.	[75]
Male Wistar rats	Administration of avocado oil (1 g/250 g weight) to diabetic rats for 90 days.	In the liver: (a) Promoted an improvement in the functionality of the electron transport chain. (b) Decreased the generation of free radicals. (c) Diminished the harmful effects of oxidative stress in the liver.	[65]
Male Wistar rats	Diet supplemented with avocado oil to rats exposed to prolonged androgenic stimulation.	Avocado oil exerted a direct regulatory effect on the lipid profile.	[76]
Wistar rats	Administration of a diet with olive oil and avocado oil to rats exposed to a diet rich in sucrose.	In the liver: (a) A diet high in sucrose causes hepatic impairment, partially reversible by the administration of avocado oil, which does not occur at the pancreatic level. (b) Avocado oil (independent of its extraction method) exhibits effects similar to those of olive oil in the fatty acid profile.	[5]
Wistar rats	Administration of a diet with olive oil and avocado oil to rats exposed to a diet rich in sucrose.	At the cardiovascular level: In rats fed with sucrose, it was observed that avocado oil reduces the levels of triglycerides, very low-density lipoprotein (VLDL) and low-density lipoprotein (LDL), without affecting the levels of high-density lipoprotein (HDL). It also reduces the level of ultrasensitive CRP, indicating that the inflammatory processes associated with metabolic syndrome are partially reestablished.	[4]
Male Wistar rats	Avocado oil administration: 1 mL of avocado oil/250 g of weight daily for a period of 90 days.	At the renal level: (a) Protection against induced oxidative stress. (b) ROS generation decrease. (c) Improvement of the activities of complexes II and III. (d) Lower peroxidizability index in diabetic mitocondria.	[67]
Male Wistar rats	The control group received a laboratory pellet, while the treated group received a diet rich in 10% avocado oil (weight/weight) for a period of two weeks.	The administration of a diet rich in avocado oil to rats for two weeks modifies the content of fatty acids in cardiac and renal membranes.	[73]
Male rabbits of the New Zealand White strain.	Rabbits were fed a semi-purified diet containing 0.2% cholesterol and 14% oil for 90 days (corn, coconut, olive and avocado).	The atherogenic power of avocado oil is equivalent to that of olive oil and lower than that of corn and coconut oil.	[72]
Female rats and chicks.	Rats and chicks were fed 10% avocado oil (*w*/*w*).	Rats and chickens fed unrefined avocado oil showed a significant decrease in total collagen solubility in the liver.	[68]
Charles river female rats.	Growing rats were fed for eight weeks with 10% (*w*/*w*) refined or unrefined avocado oil or soybean oil, using different extraction media.	Rats fed with unrefined avocado oil extracted with hexane from intact fruit (unsaponifiables) or avocado seed oil showed significant increases in the content of soluble collagen in the skin, although the total collagen content was not affected. Rats fed refined or unrefined soybean oils showed no effects.	[70]
Charles river female rats.	The rats were fed diets containing 10% avocado oil (*w*/*w*) for four weeks.	The consumption of avocado oil extracted from intact fruits can cause changes in metabolism in the liver. Serum alkaline phosphatase activity increased in rats fed seed oil, oil extracted with unrefined solvent of intact fruit or unsaponifiables, and aspartate aminotransferase activity increased significantly in the group fed avocado seed oil.	[69]
Rats	Application of oil mixtures in the skin of the dorsal area of rats for 15 days, treatment with a mixture of 2/3 of soybean oil and 1/3 avocado in a 5% solution of sweet almond oil.	The mixture of soybean and avocado oils decreases the degree of collagen cross-linking, delaying wound healing.	[71]

## Abbreviations

UAAE	Ultrasound-assisted aqueous extraction
sCO_2_	Subcritical CO_2_
scCO_2_	Supercritical CO_2_
LPG	Liquefied gas of compressed oil
FFA	Free fatty acid
MUFA	Monounsaturated fatty acid
PUFA	Polyunsaturated fatty acid
SFA	Saturated fatty acid
TAG	Triacylglycerol
O	Oleic acid
P	Palmitic acid
L	Linoleic acid
COX	Cyclooxygenase enzyme
PCR	C reactive protein
FTIR	Infrared spectroscopy with Fourier transform
IOC	International olive council
DPPH	2,2-Diphenyl-1-picrylhydrazyl
APMAE	Atmospheric pressure microwave-assisted liquid–liquid extraction
PHAs	Polyhydroxyalkanoates
ECN48	48 equivalents of carbon atoms
VLDL	Very low-density lipoprotein
LDL	Low-density lipoprotein
HDL	High-density lipoprotein
